# A Multichannel CT and Radiomics-Guided CNN-ViT (RadCT-CNNViT) Ensemble Network for Diagnosis of Pulmonary Sarcoidosis

**DOI:** 10.3390/diagnostics14101049

**Published:** 2024-05-18

**Authors:** Jianwei Qiu, Jhimli Mitra, Soumya Ghose, Camille Dumas, Jun Yang, Brion Sarachan, Marc A. Judson

**Affiliations:** 1 GE HealthCare, Niskayuna, NY 12309, USA; jianwei.qiu@gehealthcare.com (J.Q.); soumya.ghose@gehealthcare.com (S.G.); brion.sarachan@gehealthcare.com (B.S.); 2 Department of Medical Imaging, Albany Medical College, Albany, NY 12208, USA; dumasc@amc.edu (C.D.); yangj8@amc.edu (J.Y.); 3 Department of Medicine, Albany Medical College, Albany, NY 12208, USA; judsonm@amc.edu

**Keywords:** pulmonary sarcoidosis, chest CT, radiomics, ensemble network, vision transformer, CNN

## Abstract

Pulmonary sarcoidosis is a multisystem granulomatous interstitial lung disease (ILD) with a variable presentation and prognosis. The early accurate detection of pulmonary sarcoidosis may prevent progression to pulmonary fibrosis, a serious and potentially life-threatening form of the disease. However, the lack of a gold-standard diagnostic test and specific radiographic findings poses challenges in diagnosing pulmonary sarcoidosis. Chest computed tomography (CT) imaging is commonly used but requires expert, chest-trained radiologists to differentiate pulmonary sarcoidosis from lung malignancies, infections, and other ILDs. In this work, we develop a multichannel, CT and radiomics-guided ensemble network (RadCT-CNNViT) with visual explainability for pulmonary sarcoidosis vs. lung cancer (LCa) classification using chest CT images. We leverage CT and hand-crafted radiomics features as input channels, and a 3D convolutional neural network (CNN) and vision transformer (ViT) ensemble network for feature extraction and fusion before a classification head. The 3D CNN sub-network captures the localized spatial information of lesions, while the ViT sub-network captures long-range, global dependencies between features. Through multichannel input and feature fusion, our model achieves the highest performance with accuracy, sensitivity, specificity, precision, F1-score, and combined AUC of 0.93 ± 0.04, 0.94 ± 0.04, 0.93 ± 0.08, 0.95 ± 0.05, 0.94 ± 0.04, and 0.97, respectively, in a five-fold cross-validation study with pulmonary sarcoidosis (n = 126) and LCa (n = 93) cases. A detailed ablation study showing the impact of CNN + ViT compared to CNN or ViT alone, and CT + radiomics input, compared to CT or radiomics alone, is also presented in this work. Overall, the AI model developed in this work offers promising potential for triaging the pulmonary sarcoidosis patients for timely diagnosis and treatment from chest CT.

## 1. Introduction

### 1.1. Background and Motivation

Pulmonary sarcoidosis is a multisystem granulomatous interstitial lung disease (ILD) with variable presentation and prognosis. Although the disease may involve any organ, the lung is most commonly involved, at a rate of 90% in most series [1,2]. On average, a diagnosis of pulmonary sarcoidosis is made after 3 months of symptoms. In 20% of cases, pulmonary sarcoidosis patients experience symptoms up to 12 months before a diagnosis is made [3]. Currently, the diagnosis of ILD relies on a multidisciplinary approach which includes three major components—clinical presentation, chest imaging, and lung histologic findings [4,5,6,7]—wherein, both clinically and radiologically, the disease may mimic malignancies and infections [8,9,10]. Although chest-trained radiologists are familiar with the radiographic manifestations of pulmonary sarcoidosis, geographically remote and underserved locations may not have access to such radiologists. Therefore, increasing the speed and diagnostic accuracy of pulmonary sarcoidosis using imaging features has great potential to improve clinically important outcomes by directing these patients to expert care in a timelier fashion.

Both the chest radiograph and chest CT may be used to evaluate for pulmonary sarcoidosis. However, the chest CT scan is vastly superior to the chest radiograph in this regard. Several chest CT scan features are regarded as highly specific for pulmonary sarcoidosis [11,12,13], and these are often undetectable on the chest radiograph. Although such chest CT features of pulmonary sarcoidosis are regarded as highly specific for the disease and their diagnostic power was demonstrated in small cohorts [14,15], they have not been formally tested in diverse populations. Currently, there is no algorithmic diagnostic tool available that can leverage the characteristic CT findings of pulmonary sarcoidosis other than clinical diagnostic algorithms or guidelines [7,16].

### 1.2. Related Research and Gaps

Recent studies have shown that use of AI has significantly increased the efficiency of pulmonologists to distinguish respiratory diseases identified on chest CT or radiographs [17,18,19] with a limited body of research on diagnosing pulmonary sarcoidosis from chest radiographs [20,21]. There is also increasing evidence AI has the potential to democratize radiology by enabling less-experienced radiologists in underserved areas to tap into sub-specialty expertise [22]. Therefore, the development of an AI algorithm that can reliably diagnose pulmonary sarcoidosis at the level of a sub-specialty thoracic radiologist from CT would be a major advancement, an incredible asset to underserved regions, and could serve as a valued assistant for any radiologist.

Radiomics have been used extensively to build methods to automatically diagnose lung diseases and characterize lung nodules (benign vs. malignant) from CT [23,24,25,26,27,28,29]. Radiomics are hand-crafted features/mathematical descriptors extracted from radiology images that are relatively straightforward to define, conceptualize, and interpret, and are both standardized and reproducible. These features are used to train a machine learning classifier, and predictions are made based on the trained model. Specifically, radiomics and machine learning approaches have been used to classify or diagnose ILDs [30,31,32,33,34]. On the other hand, CNNs have an inherent capability to learn discriminative features within convolutional blocks for the diagnosis and classification of lung diseases [35,36,37]. These features are abstract, and it is often difficult to interpret multiscale features that are learned automatically. CNN features are unique to each input dataset, which allows considerable versatility but also introduces susceptibility to overfitting and lack of reproducibility [38].

The extraction of radiomic features typically involves defining a precise region of interest, which is difficult for diffuse lung diseases such as ILDs and pulmonary sarcoidosis, while CNNs operate on entire images or sub-images. Attempts to combine both radiomics and CNNs also have been made in several ways. For example, radiomic features were extracted from CT image and used in a deep learning network, which is an example of early fusion, and then further combined with clinical features at a late stage for the prediction of the EGFR gene mutation status for non-small cell lung carcinoma [39]. Similarly, radiomics features were extracted separately and then combined with features derived from CNN and fused at an intermediate stage before the classification of COPD staging [40] and lung nodule classification [28,41]. All methods that extracted radiomic features, however, depended on defining a region of interest, except in Liang et al. [42], where radiomics features were extracted from the entire lung, although the lung parenchyma was segmented. A comprehensive review of methods involving CNN and/or radiomics for ILDs is provided in Barnes et al. [43].

With more recent advancements in deep learning, vision transformers (ViTs) [44] have become popular in building robust classification models, sometimes outperforming CNNs [45,46]. The multiheaded, self-attention mechanism in ViT learns rich representations between the sequence of image patches, thus capturing global representation of an image. However, ViT requires large number of labeled images to train, limiting its application in studies with deficient data, particularly with medical imaging data. ViT also emphasizes low-resolution features because of the consecutive downsampling, and this results in the lack of detailed localized information [47]. On the other hand, due to strong inductive bias, CNNs can learn localized features such as edges, corners, and shapes, which may be common across different images, and can often achieve good performance with fewer training samples compared to ViT. To address the limitations of the CNN or ViT frameworks, a recent trend is to combine the ViT and CNN to sample both global and local information in an image for improved classification and segmentation tasks [48,49,50,51,52]. This combination is important for differentiating between diffuse lung diseases such as pulmonary sarcoidosis and others, as there may be some similarity in the local features of the diseases—but the relative position where the features appear within the lung becomes an important differentiating factor in classification of the disease.

### 1.3. Contributions and Novelty

Based on the distinct advantages of using radiomics, CNN or ViT, in this work, we present a novel approach using radiomics and a CT-guided multichannel CNNViT ensemble classification framework to classify pulmonary sarcoidosis vs. lung cancer (LCa). The novel aspects in our framework are as follows:

- Combination of 3D CNNs with 3D ViT that will allow capturing local information within convolutional blocks and the complex relationship between spatial positions of patches within a CT volume.
- Extraction of radiomic texture features from the chest CT without defining any region of interest, and introducing the multichannel CNNViT network architecture with a radiomic texture map and the CT volume as inputs, thus referring to the framework as RadCT-CNNViT.
- Our framework also provides visual explainability for the classification of pulmonary sarcoidosis vs. lung malignancies (LCa) that suggests regions of interest that are considered important by the network for making the prediction.

Finally, an ablation study is performed to show that our method can leverage the strengths of both hand-crafted radiomics, CT imaging features, and learned CNN+ViT features to provide improved prediction performance compared to a CNN or ViT alone, and radiomics or CT alone.

## 2. Materials and Methods

In this section, we provide overviews of the data collection process and the preprocessing steps involved in the development of our method. We subsequently explore the multichannel ensemble AI framework comprising a CNN and a ViT architecture, the extraction of radiomic texture features and combining the CNN and ViT architectures for classification. Additionally, we describe the details of the methods utilized to generate visual explanations based on the model predictions. Finally, we discuss the metrics used to evaluate the performance of the presented methods.

### 2.1. Data and Pre-Processing

**Figure 1:**
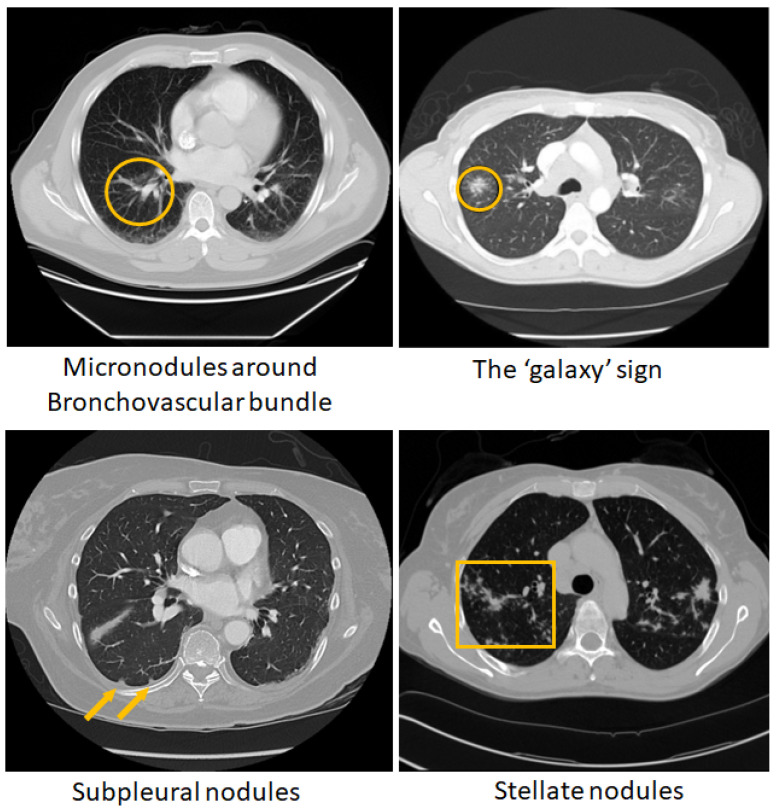
Visible patterns of pulmonary sarcoidosis on chest CT marked in ‘yellow’ circles, arrows and boxes.

The chest CT images for clinically confirmed pulmonary sarcoidosis (PS) (n = 126) were obtained from an IRB-approved study at Albany Medical College (AMC) (refer to the Compliance and Ethical Standards section for details). Chest CT exams for outpatients at AMC were performed using GE Revolution 256 CT scanner and GE VCT Lightspeed 16 slice scanner with a variety of protocols with an in-plane (*xy*) (512 × 512 matrix) resolution between 0.625 and 1 mm, and *z*-resolution of 1.25–5 mm. Patients in the pulmonary sarcoidosis database were, on average, 48.9 years of age (22–84 yrs), female (n = 77), male (n = 48), unspecified (n = 1), and white (n = 101), black (n = 17), Asian (n = 2), unspecified race (n = 6). Images of lung cancer (LCa) cases (n = 93), comprising both primary (n = 42) and metastatic (n = 51) instances, were sourced from the TCIA (LIDC-IDRI) public archive as described in previous studies [53,54]. The 3D CT volumes were center cropped in axial view to focus on the lung region, and then resized to 256 × 256 × 64. Figure 1 shows some of the chest CT patterns of pulmonary sarcoidosis.

### 2.2. The Multichannel Ensemble AI Framework for Classification

**Figure 2:**
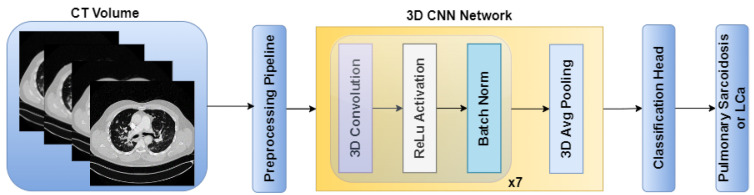
CNN architecture for pulmonary sarcoidosis vs. lung cancer (LCa) classification using chest CT images.

**Figure 3:**
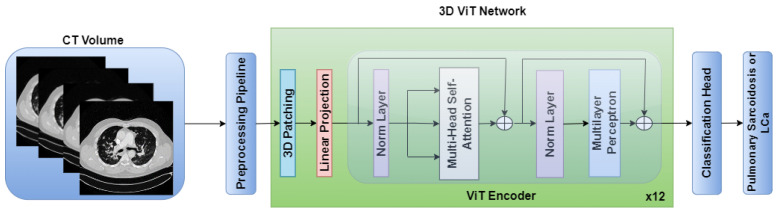
ViT architecture for pulmonary sarcoidosis vs. lung cancer (LCa) classification using chest CT images.

**Figure 4:**
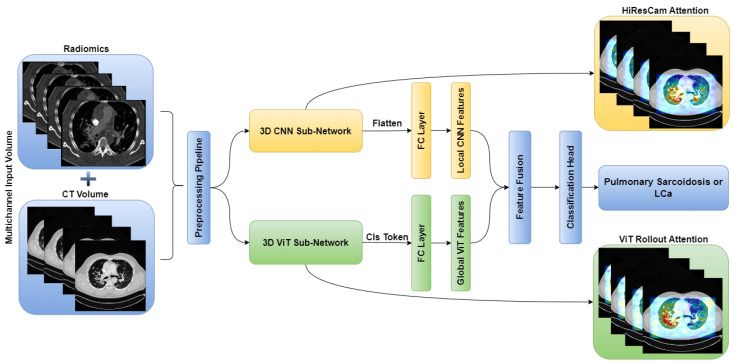
Multichannel RadCT-CNNViT architecture for pulmonary sarcoidosis vs. lung cancer (LCa) classification using chest CT images.

The standalone architectures of the CNN and ViT networks using only the CT volume as input are shown in Figure 2 and Figure 3, respectively. Subsequently, these networks are combined in an ensemble network, incorporating both the CT volume and radiomics feature, to construct the multichannel CT and radiomics-guided CNN-ViT (RadCT-CNNVIT) network. The architecture of the RadCT-CNNViT network is illustrated in Figure 4.

#### 2.2.1. Extracting Radiomics Texture

**Figure 5:**
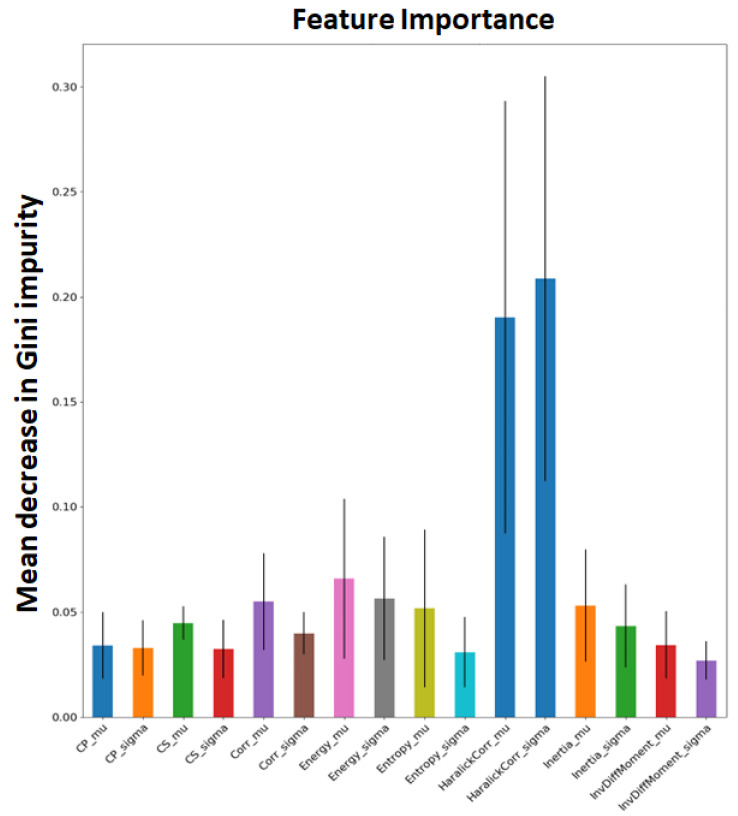
Feature importance was computed based on the mean decrease in Gini impurity for each of the Haralick texture features in discriminating pulmonary sarcoidosis from other ILDs. The mean and standard deviation of the Haralick correlation texture map were higher than those of other texture features.

**Figure 6:**
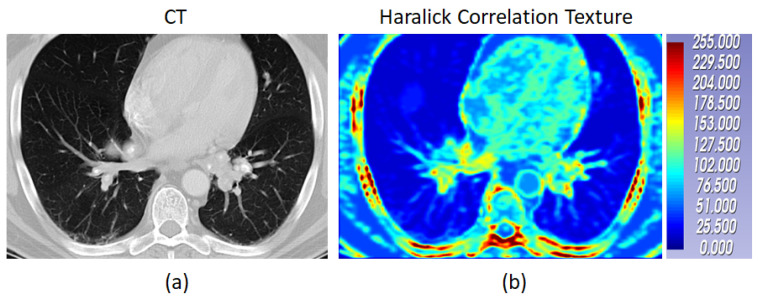
The CT of a case of pulmonary sarcoidosis and its corresponding Haralick correlation texture map are shown in (**a**) and (**b**) respectively.The color bar shows the radiomic values normalized between 0 to 255.

The input radiomics texture map for the framework was chosen based on our previous work [55], where feature selection was performed using random forest (RF) on a subset of confirmed pulmonary sarcoidosis (n = 61) and the MosMed public dataset [56] of other ILDs that were not Covid-19 (n = 154). Haralick texture features [57] such as Cluster Prominence, Cluster Shade, Correlation, Energy, Entropy, Haralick Correlation, Inertia, and Inverse Difference Moment with an offset of 1 (3 × 3 × 3 window) were computed for each CT volume and then averaged to produce one feature map per texture feature. Each radiomic texture volume was then divided into 16 × 16 × 16 patches. Patch mean and standard deviation for each of the 8 texture features were computed, resulting in a feature vector of size 16, and each patch was treated as a sample with the image label. The feature vectors from the patches were used to fit a random forest (RF) classifier [58] with 100 trees, where each patch was classified as pulmonary sarcoidosis or other ILD. The mean decrease in Gini impurity was computed as the average of feature importance scores over all trees in the RF in a 5-fold cross-validation strategy. The feature map corresponding to the highest score was chosen as input to the network architecture. Figure 5 shows all the features and their mean Gini-impurity scores after averaging across 5-folds. Figure 6 shows a case of pulmonary sarcoidosis and its corresponding Haralick correlation texture map.

#### 2.2.2. The RadCT-CNNViT Architecture

Based on Figure 5, the Haralick correlation maps were computed for pulmonary sarcoidosis and LCa cases and used as input to the RadCT-CNNViT framework with min–max intensity normalization for each 3D texture volume along with the CT volume clipped to a lung window of the (−1000, 400) intensity range. The RadCT-CNNViT is a 3D multichannel ensemble network, which consists of two input channels feeding into two subnetworks: a 3D CNN feature extractor and a 3D ViT encoder. The 3D CNN feature extractor is responsible for learning local features from the volumetric radiomic and CT feature inputs. It consists of 7 convolution blocks, where each block comprises a 3D convolution layer, ReLU activation, and batch normalization. These convolution blocks employ 3D convolutional filters to capture spatial patterns and extract relevant features from the input data. The numbers of filters in each of the convolution blocks are 16, 32, 64, 128, 256, and 512, respectively. The first convolution block utilizes a kernel size of 3 × 3 × 3 and a stride number of 1. For downsampling, the subsequent convolution blocks use a kernel size of 4 × 4 × 4 and a stride number of 2. The last layer of the 3D CNN is followed by a 3D average pooling operation and fully connected layer, which help to reduce the spatial dimensions of the CNN features to 768.

On the contrary, the 3D ViT encoder focuses on capturing global features by treating the input as a sequence of 3D patches, each with a size of 16 × 16 × 16. The 3D ViT encoder consists of 12 transformer blocks with a hidden layer dimension of 768, and each block utilizes multihead self-attention with 6 heads. The outputs from the 3D CNN feature extractor and the 3D ViT encoder are finally concatenated and fed into a fully connected (FC) layer with sigmoid activation for the classification of pulmonary sarcoidosis vs. LCa. Binary cross-entropy was used as a loss function with AdamW optimization; a learning rate of 1 × ${\mathrm{10}}^{-5}$ and 50 epochs were used to train the network. Additionally, we utilized random flip, random noise, and random affine transformations from TorchIO [59], a Python library designed for medical imaging augmentation, to augment the 3D data during training. An overview of the combined CNN-ViT network architecture is shown in Figure 4.

### 2.3. Generating Visual Explanations for Predictions

To generate visual explanations and enhance the interpretability of our model’s predictions, we applied two techniques: HiResCAM [60] and Attention Rollout [61]. These methods offer crucial insights by generating visual attention maps for both CNN and ViT sub-networks, particularly beneficial for understanding complex deep learning models applied to medical imaging, such as chest CT scans. The overarching goal is to localize relevant disease features within the chest CT volume.

HiResCAM utilizes attention mechanisms to selectively weigh the contributions of different features within the CNN subnetwork. The computation of HiResCAM is described by Equation (1). The process begins by computing the gradient of the raw score $s_{m}$ corresponding to class *m* with respect to a specific CNN feature map *A*. This gradient, represented as $\frac{\partial s_{m}}{\partial A}$, highlights the significance of various features in influencing the prediction. Subsequently, an attention map is generated by element-wise multiplication between the computed gradient and the CNN feature map, followed by summation over the feature dimension *F*. This attention map $\tilde{A}$ provides visual cues, aiding in the localization of relevant disease features within the chest CT volume:

(1) $${\tilde{A}}_{m}^{HiResCAM}=\sum_{f=1}^{F}\frac{\partial s_{m}}{\partial A}⊙A^{f}$$

In contrast, Attention Rollout offers a distinct approach by tracing the path of attention from an initial region of interest to all other patches in the image. This recursive method dynamically visualizes how the ViT sub-network distributes its attention across different parts of the image. By quantifying the attention flow, Attention Rollout provides profound insights into how the ViT sub-network distributes its attention across various parts of the image, facilitating a deeper understanding of the underlying mechanisms driving predictions. The computation of Attention Rollout at layer *L* is described by Equation (2), where $A_{L}$ represents the average of the multihead self-attention matrix at layer *L*, and *I* denotes the identity matrix:

(2) $$AttentionRollout_{L}=\left(A_{L}+I\right)AttentionRollout_{L-1}$$

### 2.4. Performance Metrics

The performance metrics for evaluation of all methods in this ablation study included sensitivity, specificity, precision, accuracy, F1-score, and combined AUC, computed across 5-folds of cross-validation. These metrics were computed based on a confusion matrix which contains four parameters: *TP* (true positive), *TN* (true negative), *FP* (false positive), and *FN* (false negative). *TP* indicates correctly predicted pulmonary sarcoidosis, *TN* denotes correctly predicted LCa, *FP* represents incorrectly predicted pulmonary sarcoidosis, and *FN* indicates incorrectly predicted LCa. Sensitivity, specificity, precision, accuracy, and F1-score values were derived from these parameters using Equations (3)–(7):

(3) $$Sensitivity=Recall=\frac{TP}{TP+FN}$$

(4) $$Specificity=\frac{TN}{TN+FP}$$

(5) $$Precision=\frac{TP}{TP+FP}$$

(6) $$Accuracy=\frac{TP+TN}{TP+TN+FP+FN}$$

(7) $$F1-Score=2*\left(\frac{Precision*Recall}{Precision+Recall}\right)$$

## 3. Experiments and Results

**Figure 7:**
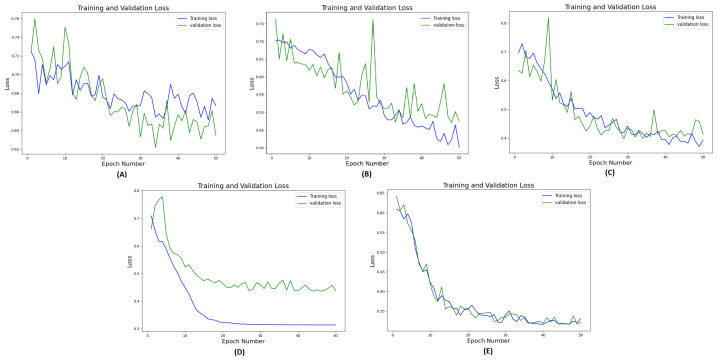
Training and validation loss curves (one-fold) for 50 epochs for the methods in ablation study: (**A**) CT-ViT, (**B**) CT-CNN, (**C**) CT-CNNViT, (**D**) Rad-CNNViT, (**E**) RadCT-CNNViT.

**Figure 8:**
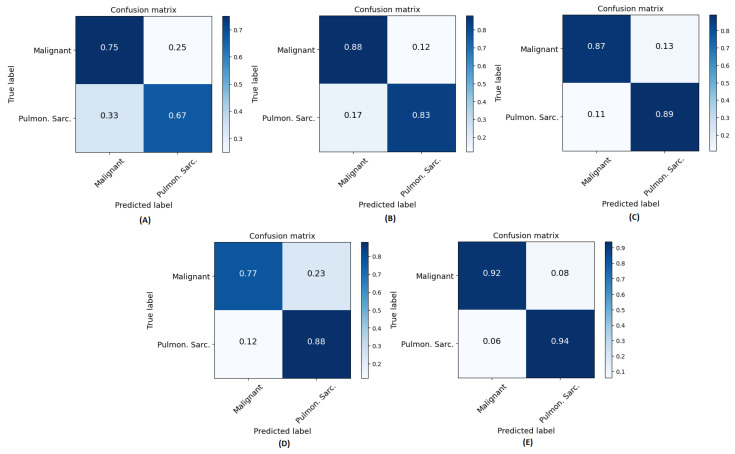
Normalized confusion matrices for all methods across all folds: (**A**) CT-ViT, (**B**) CT-CNN, (**C**) CT-CNNViT, (**D**) Rad-CNNViT, and (**E**) RadCT-CNNViT. ‘Pulmon. Sarc’. in axes labels is the abbreviation for pulmonary sarcoidosis and ‘malignant’ relates to LCa.

**Figure 9:**
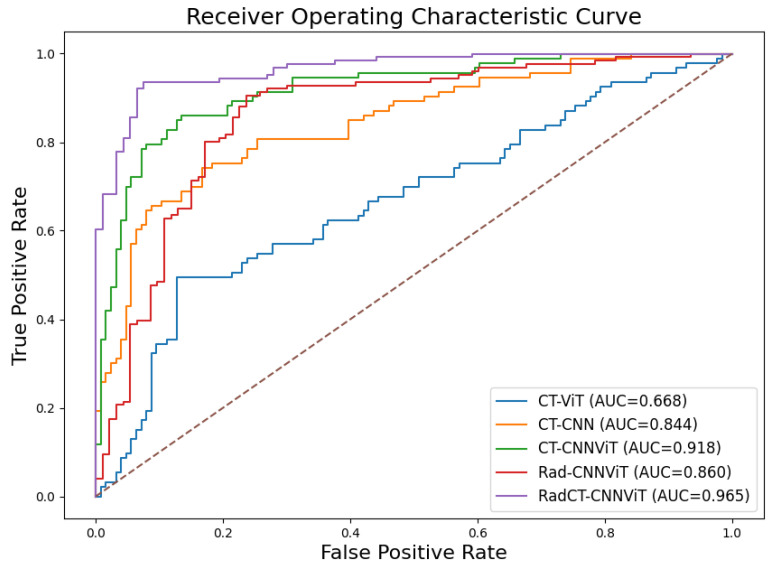
Combined receiver operating characteristic (ROC) curves for CT-ViT, CT-CNN, CT-CNNViT, Rad-CNNViT, and RadCT-CNNViT. The dotted, diagonal line represents the ROC curve for random guess.

We conducted a comprehensive ablation study to evaluate the performance of different network architectures (CNN, ViT, and CNNViT) using CT, radiomics, and multichannel CT-radiomics data. In this study, we performed a five-fold cross-validation with a dataset of clinically confirmed cases of pulmonary sarcoidosis (n = 126) and lung cancer (n = 93). Figure 7 illustrates the training and validation loss curves of a single fold over 50 epochs for all different methods compared. It demonstrates that 3D ViT failed to converge due to the limited training dataset, while 3D CNN showed slower convergence with unstable loss. Conversely, 3D CNN-ViT ensemble network demonstrated improved convergence due to the combination of global and local features. Moreover, RadCT-CNNViT achieved the lowest loss and best converged training and validation losses in differentiating the diseases, further demonstrating the effectiveness of leveraging radiomics texture maps as input along with CT. The normalized confusion matrices for all experiments in this ablation study are shown in Figure 8. The confusion matrices show that the true prediction rate for pulmonary sarcoidosis increased higher than LCa when the CNN and ViT networks were combined, suggesting the value of combining global and local features for pulmonary sarcoidosis. Performance metrics for these experiments were derived from the confusion matrices and are summarized in Table 1. Additionally, the corresponding ROC curves are depicted in Figure 9. The RadCT-CNNViT model demonstrated the best performance, with accuracy, sensitivity, specificity, precision, F1-score, and combined AUC of 0.93 ± 0.04, 0.94 ± 0.04, 0.93 ± 0.08, 0.95 ± 0.05, 0.94 ± 0.04 and 0.97, respectively, compared to other variations in the ablation study, with statistical significance of p<0.0001.

**Figure 10:**
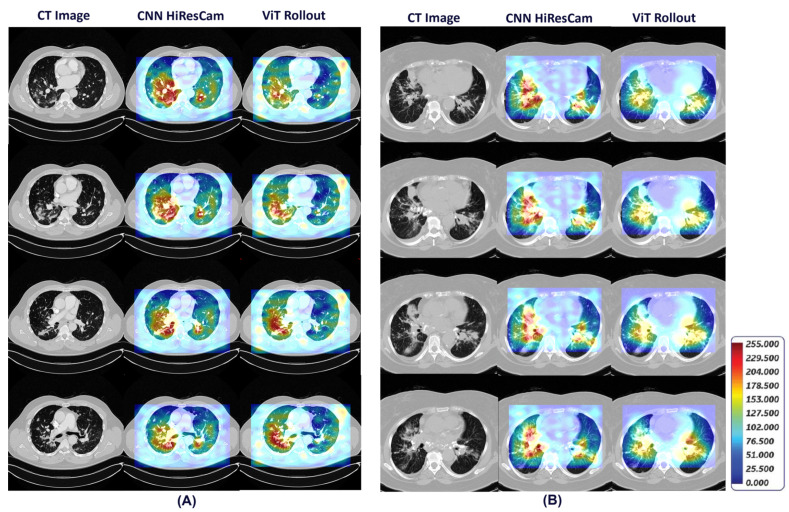
HiResCAM and ViT Attention Rollout visual explanations that highlight the regions of interest on CT scan associated with diagnosis of pulmonary sarcoidosis (**A**) and lung cancer (**B**).

Figure 10 shows detailed visual explanations utilizing HiResCAM and ViT Attention Rollout techniques for both pulmonary sarcoidosis and LCa. The computed visual attention maps are overlaid onto the CT images to emphasize the regions of interest. We observed that features from the CNN subnetworks had denser visual representations (color maps) within local regions, while ViT showed overall global representations as expected. These visual cues highlight features in specific regions of interest contributing to pulmonary sarcoidosis and lung cancer diagnoses.

## 4. Discussion

We presented a method to diagnose pulmonary sarcoidosis from LCa through a combination of CNN and ViT in two parallel branches of the network, retaining both local and global representations, along with a radiomics map as an additional input channel with CT volume. Although there have been previous attempts to combine CNN and ViT in various forms for disease diagnosis, we believe this is one of the first use cases of using radiomics texture maps and CT as 3D volumetric, multichannel inputs in a CNN-ViT framework. Previous studies have typically shown a combination of radiomics and CNN features for lung disease classification, prognosis and staging using late fusion techniques, i.e., radiomics features and CNN features were combined just before the classification layer, which demonstrated improved performance compared to CNN features or radiomics features based classification only [39,40,62]. In our previous work [55], we showed how radiomics texture features used as input to a CNN-ViT framework had improved performance over using radiomics features with a traditional machine learning classifier to classify pulmonary sarcoidosis from other ILDs.

In this work, we show that compared to a CNN or ViT alone, or using CT or radiomics only in a CNN-ViT ensemble network, a CT and radiomics-guided deep learning approach provides improved feature representation. Specifically, it highlights the effectiveness of feature fusion, in both early and intermediate stages, i.e., the proper utilization of radiomics texture maps, which are also 3D volumes extracted from CT as input features along with CT imaging features, and combining features extracted from CNN and ViT sub-networks before classification. The strong inductive bias of CNNs is necessary to reach the desired classification accuracy with less data. However, for diffuse lung diseases with no specific location within the lung, the global, long-range context offered by ViT is more adept at identifying/embedding interactions between image patches. Unfortunately, ViT does not provide as much local context compared to CNNs. Nevertheless, the problem of precisely embedding the local and global representations into one another remains. Hence, in this work, a dual structure of CNN-ViT is created to capture the respective feature representations for enhanced representation learning.

Radiomics texture features are computationally well defined compared to abstract, hierarchical, and difficult-to-interpret CNN features. Haralick texture (correlation map), used in this work, captures features from the CT images that are not perceptible for the human eye [63]. In essence, it describes how often one gray tone will appear in a specified spatial relationship to another gray tone on the image [64]. As a result, subtle differentiation between different granulomatous disorders such as between the ‘galaxy sign’ of pulmonary sarcoidosis and that mimicking metastatic lung cancer [65,66] is possible using such radiomics texture features. Our experiments showed that although the inclusion of radiomics feature as multichannel input with CT did improve all performance metrics in differentiating pulmonary sarcoidosis from LCa, neither CT nor radiomics alone could provide similar accuracies, leading to the confirmation of the hypothesis that the radiomics texture indeed was complementary to CT imaging features. However, we acknowledge that an extensive set of radiomics texture maps was not computed in our experiments, and only the Haralick textures were computed, which is a limitation of this work. The types of radiomics texture features are myriad, and the computation of all features, down-selecting the best features to remove noisy representations, is an intensive process. In future, we plan to include transform-based texture features in our experiments. The radiomics texture map extraction for pulmonary sarcoidosis or LCa did not involve the annotation of regions of interest in the CT volume, which makes our AI framework further suitable for differentiating between other types of diffuse lung diseases.

The major limitations of this work include training and validation using a cross-validation approach due to the limited sample size, and unavailability of a separate validation set from a multicenter study, which may affect the generalizability of the method. However, this being a pilot study to choose the best performing method between the radiomics, CNN, and ViT combinations, the improvement of one method over the other is observed in the results without testing on a separate cohort. Additionally, our method only addresses a two-class problem, i.e., diagnosing pulmonary sarcoidosis vs. LCa; however, in clinical settings, differentiating between pulmonary sarcoidosis and other forms of ILDs would be necessary, which is part of our future work.

We also acknowledge that the presented RadCT-CNNViT is complex in terms of training the network, as it requires a GPU compute; we do not, however, think this limits the adoption of the method in a clinical setting, as, based on our experience, 3D network inferences can be often performed on CPUs with advanced Intel optimization techniques. Additionally, we used a vanilla CNN and a standard ViT network in our implementation without trying different CNN or Vision Transformer versions such as ResNets or Swin Transformers because of the limited training sample size, as complex deep learning networks need a lot more data for model convergence during training. Applying individually pretrained CNNs or ViTs to mitigate the limited training data issue was not in the scope of this work, as pretrained networks are 2D and a variety of approaches for transfer learning may be taken into consideration for 3D medical images, and choosing the right strategy for utilizing such models for disease diagnosis depends on network design choices, and on the similarity and the amount of the dataset [67]. One of the hypotheses of this work was to show that while ViTs provide rich global feature representations, they do not outperform CNNs in a low-data setting, and by combining CNN and ViT, a reasonably acceptable classification performance can be achieved. In addition, the combination of local and global features is clinically relevant in the diagnosis of pulmonary sarcoidosis. Although, there is no prior literature on the sensitivity and specificity of diagnosing pulmonary sarcoidosis from chest CT, prior work [15] suggests that the performance of our method is similar to that of expert radiologists in diagnosing cardiac sarcoidosis with pulmonary and mediastinal involvement. The performance of our method is also higher than previous works that used chest X-ray to diagnose pulmonary sarcoidosis from healthy patients [21] or patients with pneumonia [20] involving deep learning or radiomics respectively with much smaller cohorts.

## 5. Conclusions

Pulmonary sarcoidosis is a diffuse lung disease, which is difficult to diagnose from CT imaging without a multidisciplinary clinical team, specifically in geographically underserved locations. The visual attention maps and intelligent network architecture from CNN and ViT used in our method are likely to reduce the burden of radiologists and provide a timely and reliable probability of pulmonary sarcoidosis diagnoses. Clinicians may also use this information directly to adjust their diagnostic probabilities in patients with diffuse lung disease. As our AI method to diagnose pulmonary sarcoidosis does not depend on input from radiologists, it may truly augment the radiologist’s impression, as the approaches of the radiologist and our method most probably will be different. This suggests that our method may not only increase the speed of the radiographic assessment of diffuse lung disease but may surpass current chest imaging diagnostic standards. Finally, although our method was applied to pulmonary sarcoidosis in this instance, it could be adapted to any interstitial lung disease. We therefore believe that our method ultimately has the capability to be used as a general diagnostic tool for all interstitial lung diseases as well as localized lung diseases.

## Figures and Tables

**Table 1:** Performance statistics (Sensitivity, Specificity, Accuracy and combined area under curve (AUC)) for CT-ViT, CT-CNN, CT-CNNViT, Rad-CNNViT, and RadCT-CNNViT.

Network	Sensitivity	Specificity	Precision	Accuracy	F1-Score	AUC
CT-ViT	0.68 ± 0.09	0.66 ± 0.02	0.72 ± 0.08	0.67 ± 0.05	0.70 ± 0.08	0.67
CT-CNN	0.83 ± 0.04	0.88 ± 0.05	0.89 ± 0.06	0.85 ± 0.04	0.86 ± 0.05	0.84
CT-CNNViT	0.87 ± 0.05	0.89 ± 0.06	0.92 ± 0.05	0.88 ± 0.04	0.89 ± 0.05	0.92
Rad-CNNViT	0.88 ± 0.06	0.77 ± 0.09	0.84 ± 0.06	0.84 ± 0.05	0.86 ± 0.06	0.86
RadCT-CNNViT	**0.94 ± 0.04**	**0.93 ± 0.08**	**0.95 ± 0.05**	**0.93 ± 0.04**	**0.94 ± 0.04**	**0.97**

Bold values indicate best performance.

## Data Availability

Retrospective data for pulmonary sarcoidosis were collected at Albany Medical College and cannot be shared publicly.
